# Author Correction: Therapy-induced secretion of spliceosomal components mediates pro-survival crosstalk between ovarian cancer cells

**DOI:** 10.1038/s41467-024-50958-x

**Published:** 2024-07-31

**Authors:** Victoria O. Shender, Ksenia S. Anufrieva, Polina V. Shnaider, Georgij P. Arapidi, Marat S. Pavlyukov, Olga M. Ivanova, Irina K. Malyants, Grigory A. Stepanov, Evgenii Zhuravlev, Rustam H. Ziganshin, Ivan O. Butenko, Olga N. Bukato, Ksenia M. Klimina, Vladimir A. Veselovsky, Tatiana V. Grigorieva, Sergey Y. Malanin, Olga I. Aleshikova, Andrey V. Slonov, Nataliya A. Babaeva, Lev A. Ashrafyan, Elena Khomyakova, Evgeniy G. Evtushenko, Maria M. Lukina, Zixiang Wang, Artemiy S. Silantiev, Anna A. Nushtaeva, Daria D. Kharlampieva, Vassili N. Lazarev, Arseniy I. Lashkin, Lorine K. Arzumanyan, Irina Yu. Petrushanko, Alexander A. Makarov, Olga S. Lebedeva, Alexandra N. Bogomazova, Maria A. Lagarkova, Vadim M. Govorun

**Affiliations:** 1 Center for Precision Genome Editing and Genetic Technologies for Biomedicine, Lopukhin Federal Research and Clinical Center of Physical-Chemical Medicine of Federal Medical Biological Agency, Moscow, 119435 Russian Federation; 2 Lopukhin Federal Research and Clinical Center of Physical-Chemical Medicine of the Federal Medical and Biological Agency, Moscow, 119435 Russian Federation; 3 Shemyakin-Ovchinnikov Institute of Bioorganic Chemistry of the Russian Academy of Sciences, Moscow, 117997 Russian Federation; 4 Faculty of Biology; Lomonosov Moscow State University, Moscow, 119991 Russian Federation; 5 Moscow Institute of Physics and Technology (State University), Dolgoprudny, 141701 Russian Federation; 6 Faculty of Chemical-Pharmaceutical Technologies and Biomedical Drugs, Mendeleev University of Chemical Technology of Russia, Moscow, 125047 Russian Federation; 7 Institute of Chemical Biology and Fundamental Medicine, Siberian Branch, Russian Academy of Sciences, Novosibirsk, 630090 Russian Federation; 8 Department of Natural Sciences, Novosibirsk State University, Novosibirsk, 630090 Russia; 9 Kazan Federal University, Kazan, 420008 Russian Federation; 10 National Medical Scientific Centre of Obstetrics, Gynaecology and Perinatal Medicine named after V.I. Kulakov, Moscow, 117198 Russian Federation; 11 Russian Research Center of Roentgenology and Radiology, Moscow, 117997 Russian Federation; 12 Exosome Analytics, Evry, 91058 France; 13 Faculty of Chemistry; Lomonosov Moscow State University, Moscow, 119991 Russian Federation; 14 Department of Obstetrics and Gynecology, Qilu Hospital, Cheeloo College of Medicine, Shandong University; Jinan, 250012 Shandong, China; 15 Engelhardt Institute of Molecular Biology, Russian Academy of Sciences, Moscow, 119991 Russian Federation; 16 Research Institute for Systems Biology and Medicine, Moscow, 117246 Russian Federation

**Keywords:** Ovarian cancer, Proteomic analysis, Proteomics, Mass spectrometry, Transcriptomics

Correction to: *Nature Communications* 10.1038/s41467-024-49512-6, published online 19 June 2024

In the Acknowledgements section, the State funding project ‘12403120004-7’ should have read ‘124031200004-7’. The original article has been corrected.

