# Supplementary material for: Therapy-induced secretion of spliceosomal components mediates pro-survival crosstalk between ovarian cancer cells
Source: Nat Commun. 2024 Jun 19;15:5237. doi: 10.1038/s41467-024-49512-6 (PMC11187153; doi:10.1038/s41467-024-49512-6)
Supplement: Supplementary file 1 — Supplementary Information [file 41467_2024_49512_MOESM1_ESM.pdf]

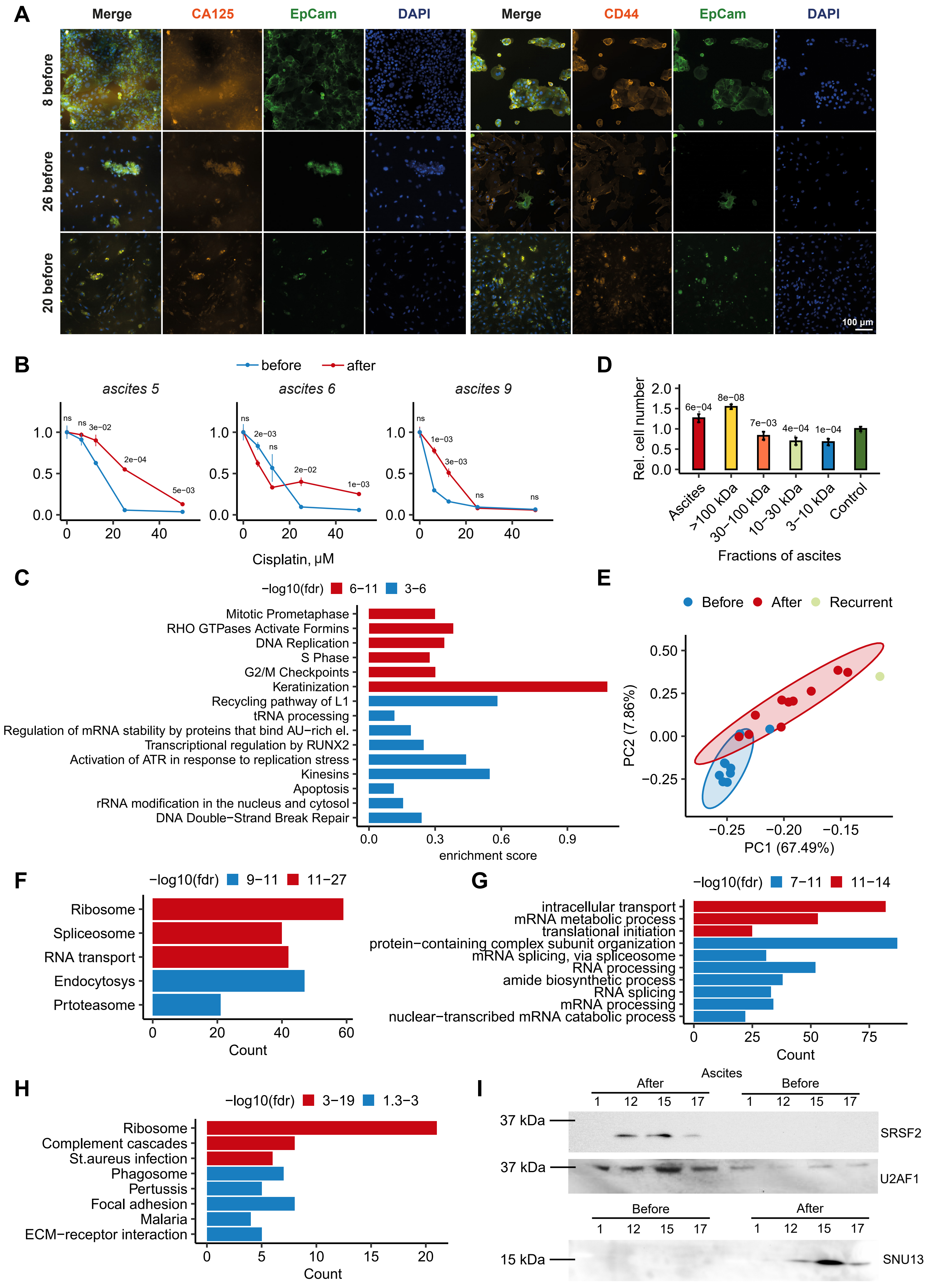
A
Merge
CA125
EpCam
DAPI
Merge
CD44
EpCam
DAPI
8 before
26 before
20 before
100 µm
B
before
after
ascites 5
ascites 6
ascites 9
ns
ns
3e−02
2e−04
5e−03
2e−03
ns
2e−02
1e−03
ns
1e−03
3e−03
ns
ns
1.0
0.5
0.0
0
20
40
Cisplatin, µM
D
Rel. cell number
2.0
1.5
1.0
0.5
0.0
6e−04
8e−08
7e−03
4e−04
1e−04
Ascites
>100 kDa
30−100 kDa
10−30 kDa
3−10 kDa
Control
Fractions of ascites
C
−log10(fdr)
6−11
3−6
Mitotic Prometaphase
RHO GTPases Activate Formins
DNA Replication
S Phase
G2/M Checkpoints
Keratinization
Recycling pathway of L1
tRNA processing
Regulation of mRNA stability by proteins that bind AU−rich el.
Transcriptional regulation by RUNX2
Activation of ATR in response to replication stress
Kinesins
Apoptosis
rRNA modification in the nucleus and cytosol
DNA Double−Strand Break Repair
0.0
0.3
0.6
0.9
enrichment score
E
Before
After
Recurrent
PC2 (7.86%)
0.50
0.25
0.00
−0.25
−0.25
−0.20
−0.15
PC1 (67.49%)
F
−log10(fdr)
9−11
11−27
Ribosome
Spliceosome
RNA transport
Endocytosys
Prtoteasome
0
20
40
60
Count
G
−log10(fdr)
7−11
11−14
intracellular transport
mRNA metabolic process
translational initiation
protein−containing complex subunit organization
mRNA splicing, via spliceosome
RNA processing
amide biosynthetic process
RNA splicing
mRNA processing
nuclear−transcribed mRNA catabolic process
0
25
50
75
Count
H
−log10(fdr)
3−19
1.3−3
Ribosome
Complement cascades
St.aureus infection
Phagosome
Pertussis
Focal adhesion
Malaria
ECM−receptor interaction
0
5
10
15
20
Count
I
Ascites
After
Before
1
12
15
17
37 kDa
SRSF2
U2AF1
15 kDa
SNU13

Supplementary Figure 1. Malignant ascites after chemotherapy promote a more aggressive phenotype of recipient ovarian cancer cells. ***(A)*** Fluorescence images of primary cultures of ovarian cancer cells stained for CA125, EpCam, and CD44. Nuclei are stained by DAPI (blue). ***(B)*** *In vitro* viability assay of ovarian cancer cells isolated from paired ascites from patients before (blue curves) and after (red curves) chemotherapy and subsequently treated with different concentrations of cisplatin. Dose-response curves of ovarian cancer cells were determined by an MTT assay on day 2 after cisplatin adding. The data represent the mean values ± SD (standard deviation) (n=3 biologically independent experiments). ***(C)*** GSEA analysis of gene expression in neuroendocrine ovarian cancer cells pre-incubated for 3 days with autologous ascites after chemotherapy versus ascites before therapy. The X-axis represents GSEA enrichment score (p-values are indicated by colors). ***(D)*** Primary ovarian cancer cell culture was pre-incubated for 3 days with different fractions of ascites from a patient after chemotherapy, and then cancer cells were treated with 10 μM of cisplatin. *In vitro* cell viability assay was performed on day 2 after cisplatin adding. All data represent the mean values ± SD (n=6 biologically independent samples). ***(E)*** The principal component analysis of proteomic profiles of paired ascites before and after chemotherapy. ***(F)*** Results of the KEGG enrichment analysis of proteins whose abundance increased at least 2 times in ovarian cancer ascites after chemotherapy compared to ovarian cancer ascites before therapy (p-values are indicated). ***(G)*** Gene Ontology enrichment analysis of proteins identified only in ovarian cancer ascites after chemotherapy (p-values are indicated by colors). ***(H)*** KEGG enrichment analysis of precursor proteins identified in ovarian cancer ascites after chemotherapy (p-values are indicated by colors). ***(I)*** Western blotting analysis of paired ascites from patients with ovarian cancer before and after chemotherapy.

The p-value was obtained by two-tailed unpaired Student's t-test (*B*). ClusterProfiler was used for functional enrichment analysis with all genes as background (*F, G, H*). Gene expression signature analysis was performed using the "signatureSearch" packages in "R" against the Reactome database (*C*). A hypergeometric test was carried out and all significant categories (false discovery rate < 0.05, after correction for multiple testing using the Benjamini–Hochberg procedure) are displayed. Source data are provided as a Source Data file.

Extended Figure 2

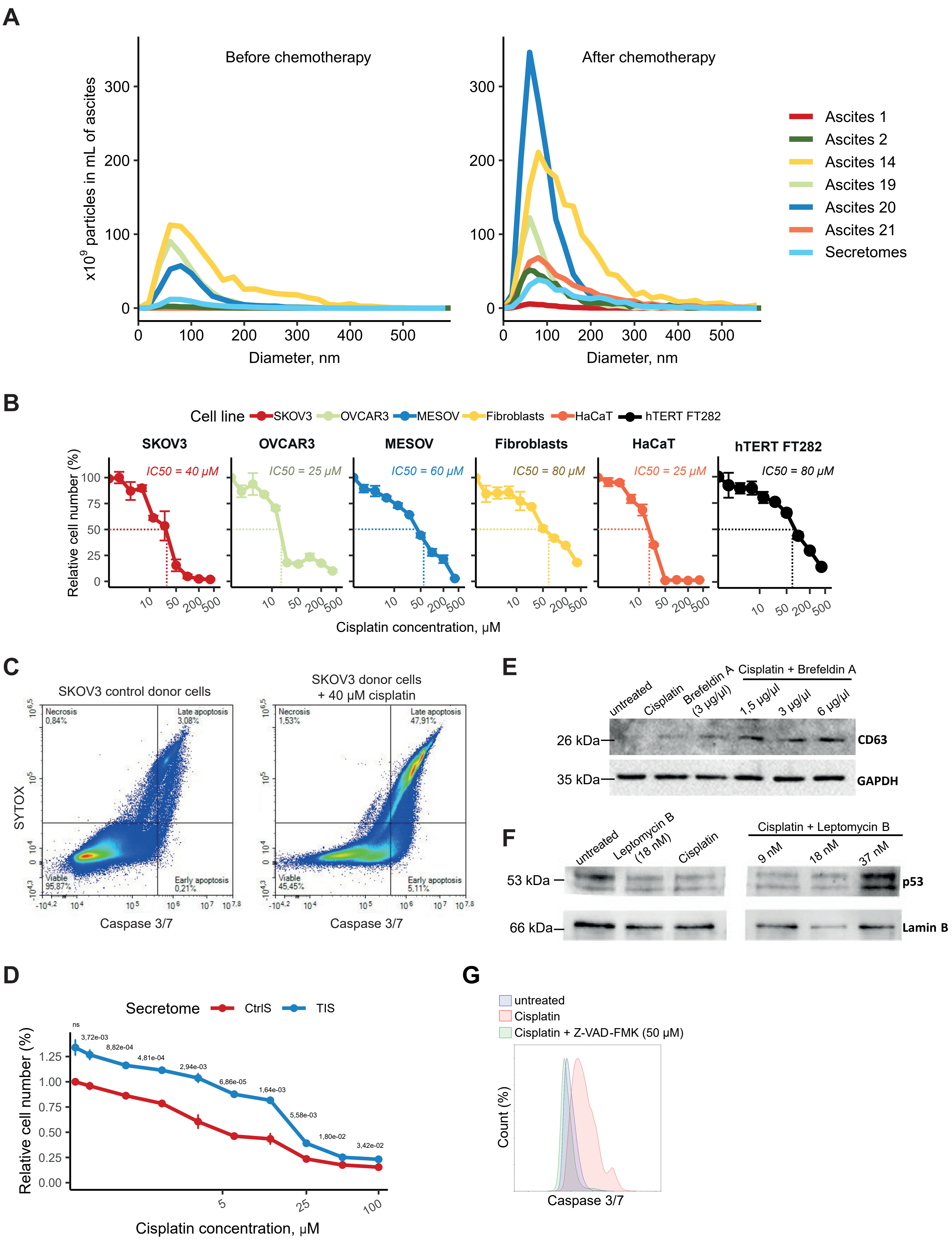

Supplementary Figure 2. Effect of therapy-induced secretomes on cancer cells. ***(A)*** Size distribution of extracellular vesicles isolated from paired ascites before and after chemotherapy, and from secretomes of SKOV3 cells treated or untreated with cisplatin (secretomes were collected as indicated in Fig. 3B) (n=14 biologically independent samples). Data represent the mean values. ***(B)*** Dose-response curves obtained by MTT assay of fibroblasts, HaCaT, hTERT FT282, OVCAR3, MESOV, and SKOV3 cells that were treated with different concentrations of cisplatin for 7 h, then cells were washed three times with PBS and cultured for 41 h. Each data point represents mean values ± SD (n=3 biologically independent experiments). The dotted lines indicate IC50 cisplatin values, which were determined by fitting a normalized model to data with nonlinear regression using GraphPad Prism software. ***(C)*** CellEvent Caspase-3/7 Green Flow Cytometry Assay of untreated SKOV3 cells and SKOV3 cells treated with 40 µM cisplatin for 7 h, and then all cells were washed three times with PBS and cultured for 41 h. ***(D)*** Dose-response curves obtained by MTT assay of recipient primary culture of ovarian cancer cells 26 that were incubated with therapy-induced (TIS) or control (CtrlS) secretomes for 3 days, and then treated with different concentrations of cisplatin for 2 days. Secretomes were obtained from donor cells 26 that were treated or untreated with cisplatin (25 µM) for 7 h, washed with PBS and cultured in serum free media for 41 h. The data represent the mean values ± SD (n=3 biologically independent experiments). ***(E and F)*** Western blot analysis of donor SKOV3 cells treated with Brefeldin A or Leptomycin B in combination with 40 µM cisplatin (cells were treated as indicated in Fig. 3D). To select a Brefeldin A effective concentration, the retention level of secreted protein CD63 was assessed inside cells ***(E)***. For a selection of Leptomycin B effective concentration, a rate of nuclear export of p53 was assessed ***(F)***. ***(G)*** Flow cytometry analysis of caspase 3/7 activity in SKOV3 cells treated with Z-VAD-FMK in combination with cisplatin (40 µM) to check an effective inhibition of caspase activity (cells were treated as indicated in Fig. 3D).

The p-value was obtained by two-tailed unpaired Student's t-test (*D*). Source data are provided as a Source Data file.

Extended Figure 3

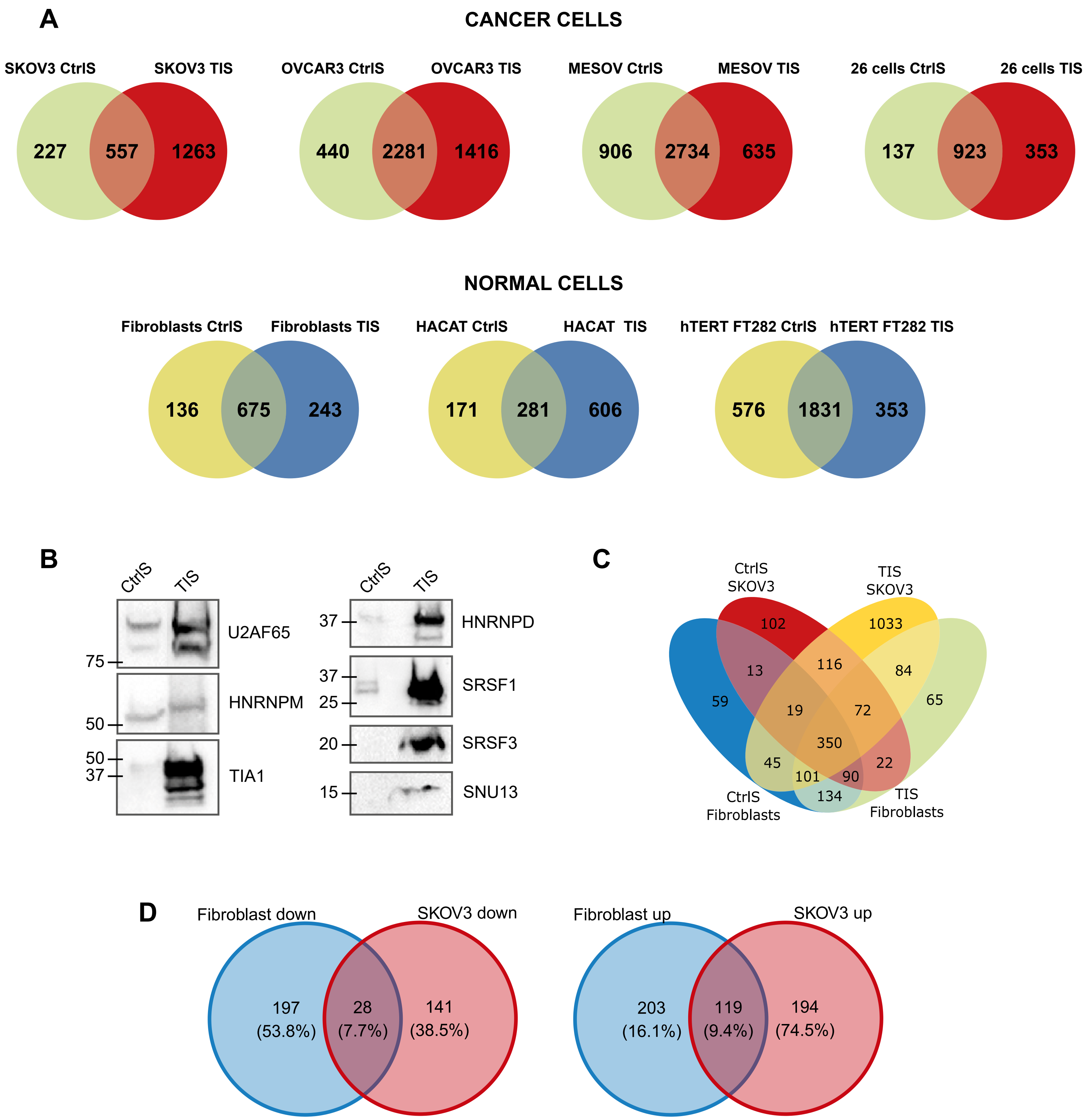

Supplementary Figure 3. Proteomic profiles of secretomes from different cell lines before and after cisplatin treatment. ***(A)*** Venn diagram representing the proteins identified in therapy-induced (TIS) and control (CtrlS) secretomes from HaCaT, hTERT FT282, SKOV3, MESOV, OVCAR3, 26 cells, and fibroblasts. ***(B)*** Western blotting analysis of secretomes from SKOV3 cells treated (TIS) or untreated (CtrlS) with cisplatin (secretomes were collected as indicated in Fig. 3B). ***(C)*** Venn diagram representing the proteins identified in therapy-induced (TIS) and control (CtrlS) secretomes from SKOV3 cells and fibroblasts. ***(D)*** Intersection of upregulated right Venn diagram) and down-regulated (left Venn diagram) proteins identified in therapy-induced secretomes from SKOV3 cells and fibroblasts versus their control secretomes. Source data are provided as a Source Data file.

Extended Figure 4

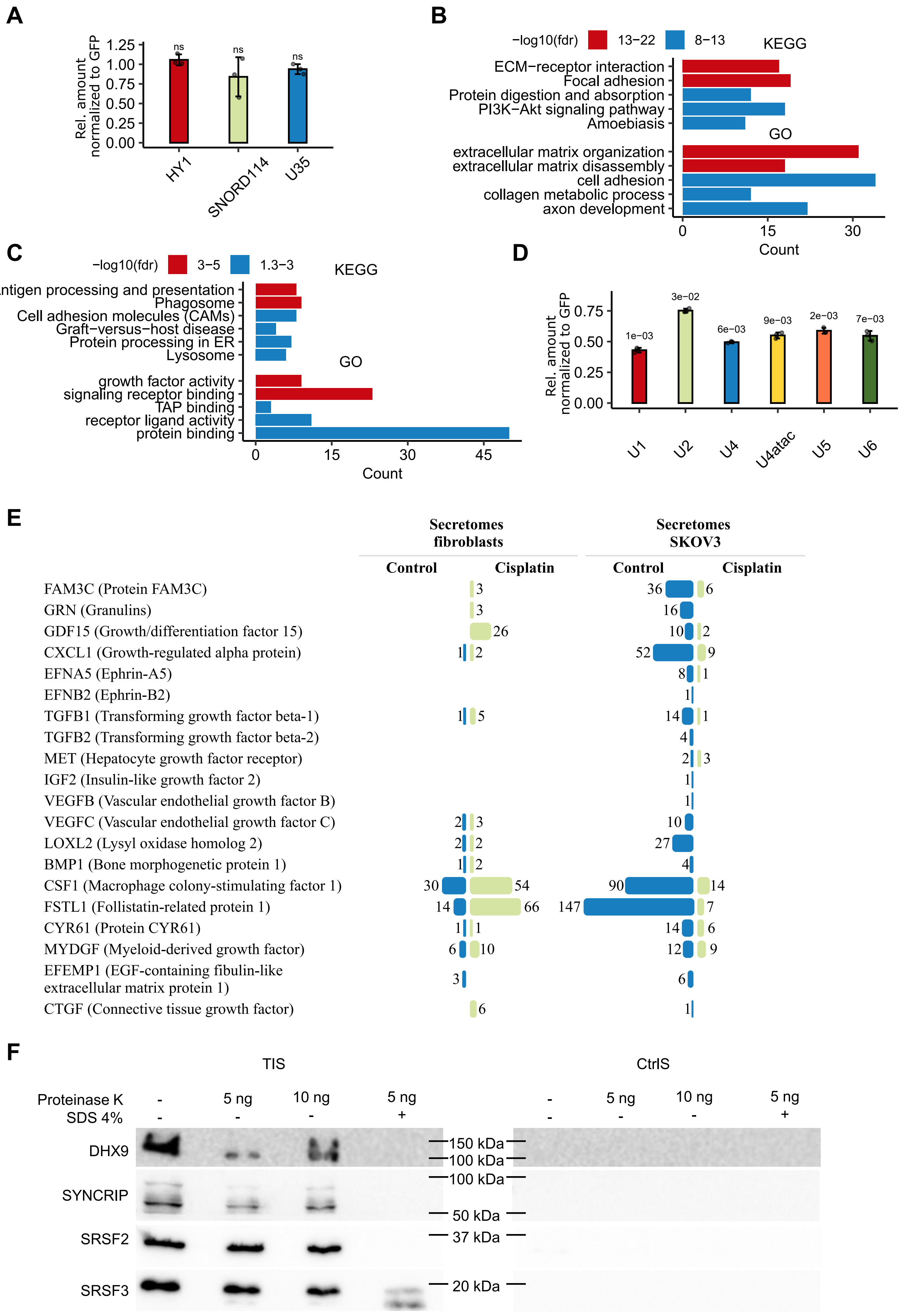

Supplementary Figure 4. Spliceosomal proteins are exported from drug-stressed cancer but not fibroblasts. ***(A)*** RT-qPCR analysis of 3 noncoding snRNAs not associated with spliceosome in therapy-induced or control secretomes of SKOV3 cells (secretomes were collected as indicated in Fig. 3B). Bars represent the level of each snRNA in therapy-induced secretomes compared to control secretomes. All data represent the mean values ± SD (n=3 biologically independent samples). ***(B)*** KEGG and Gene Ontology enrichment analysis of down-regulated proteins identified in therapy-induced secretomes from fibroblasts (p-values are indicated by colors). ***(C)*** KEGG and Gene Ontology enrichment analysis of upregulated proteins identified in therapy-induced secretomes from fibroblasts (p-values are indicated by colors). ***(D)*** RT-qPCR analysis of spliceosomal snRNAs in therapy-induced or control secretomes of fibroblasts (secretomes were collected as indicated in Fig. 3B). Bars represent the level of each snRNA in therapy-induced secretomes compared to control secretomes. All data represent the mean values ± SD (n=3 biologically independent samples). ***(E)*** Bar plot with spectral counts of selected proteins identifying in therapy-induced or control secretomes from SKOV3 cells and fibroblasts. ***(F)*** Results of proteinase K protection assay. Western blotting analysis of secretomes from SKOV3 cells treated (TIS) or untreated (CtrlS) with cisplatin (secretomes were collected as indicated in Fig. 3B) and then samples were treated or untreated with different concentrations of proteinase K in presence or absence of detergent.

The p-value was obtained by two-tailed unpaired Student's t-test (*A*, *D*). ClusterProfiler was used for functional enrichment analysis with all genes as background (*B*, *C*). A hypergeometric test was carried out and all significant categories (false discovery rate < 0.05, after correction for multiple testing using the Benjamini–Hochberg procedure) are displayed. Source data are provided as a Source Data file.

Extended Figure 5

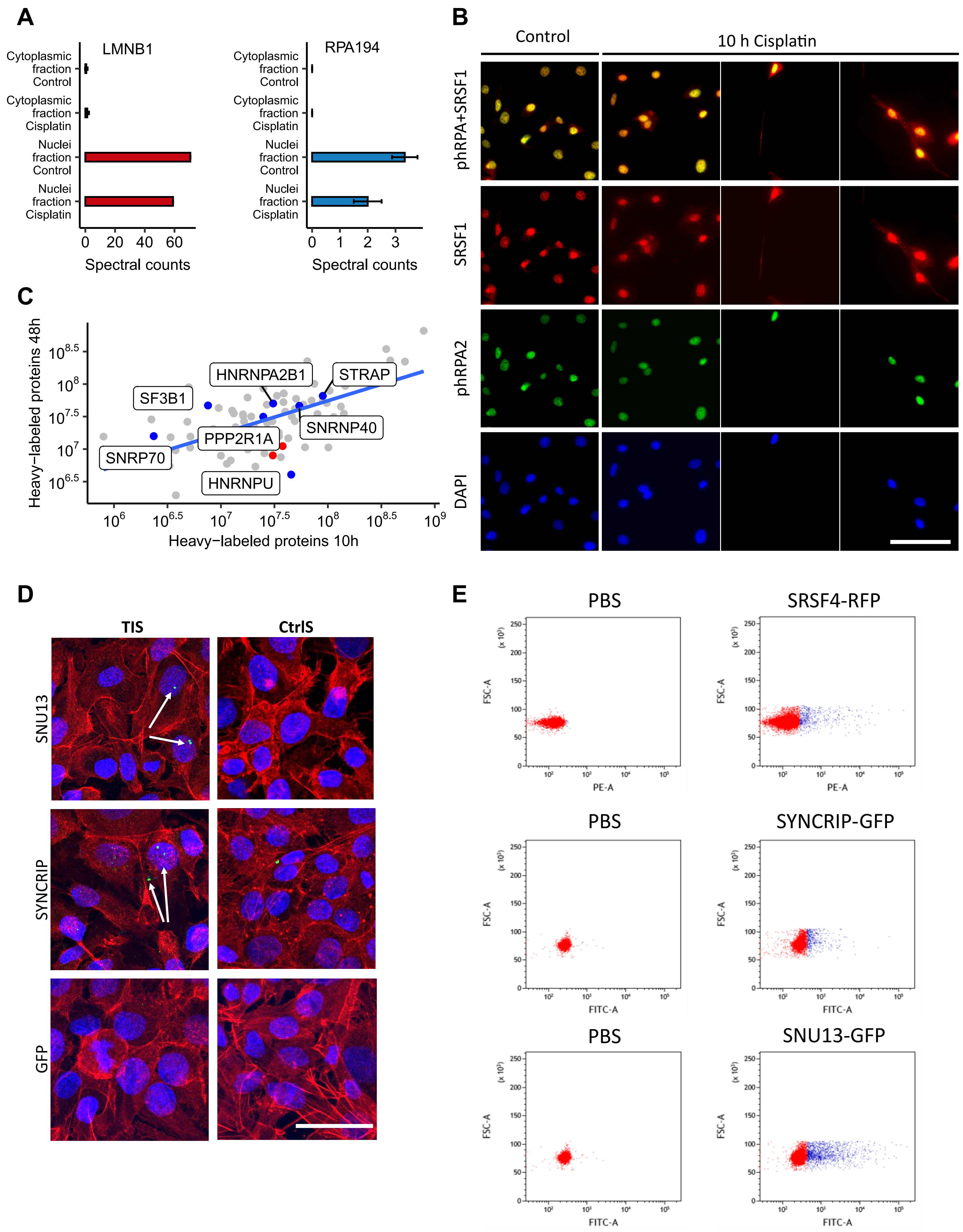

Supplementary Figure 5. Spliceosomal proteins are secreted by drug-stressed ovarian cancer cells and transferred to recipient cancer cells. ***(A)*** Spectral counts of nuclear markers (LMNB1 and RPA194) identified in nuclear and cytoplasmic fractions of SKOV3 cells before and after cisplatin treatment (as indicated in Fig. 3B). The data represent the mean values ± SD (n=2).***(B)*** Fluorescence images of SKOV3 cells stained for SRSF1 (red), pRPA2 (Ser33, green) and with DAPI (blue) before and after treatment with 40 µM cisplatin for 7 h. Scale bar is 100 µm. ***(C)*** Dot plot of LFQ intensities of heavy-labeled proteins identified in SKOV3 cells after incubation with extracellular vesicles for 10 (x-axis) and 48 h (y-axis). Dots located over a trend line indicate heavy-labeled proteins that were retained in recipient cells for 48 hours. Blue dots represent spliceosomal proteins; red dots represent ribosomal proteins. ***(D)*** Confocal immunofluorescence images depict recipient SKOV3 cells incubated for 3 days with therapy-induced secretomes (TIS) or control secretomes (CtrlS) from donor SKOV3 cells overexpressing GFP (green), SYNCRIP-GFP (green), or SNU13-GFP (green). Secretomes were collected as indicated in Fig. 3B. Actin filaments are stained with Phalloidin (red), while nuclei are stained with DAPI (blue). Scale bar: 25 µm. ***(E)*** Flow cytometry dot plots (forward side scattering (FSC) vs intensities of green fluorescence (FITC-channel) or red fluorescence (PE-channel)). Flow cytometry analysis was conducted for magnetics beads coated with anti-CD9 antibody after immunoprecipitation of extracellular vesicles from therapy-induced secretomes of donor SKOV3 cells. Donor SKOV3 cells overexpressing SRSF4-RFP, SYNCRIP-GFP or SNU13-GFP were treated with 40 µM cisplatin (as indicated in Fig. 3B). High-fluorescent events are highlighted in blue. Source data are provided as a Source Data file.

Extended Figure 6

A

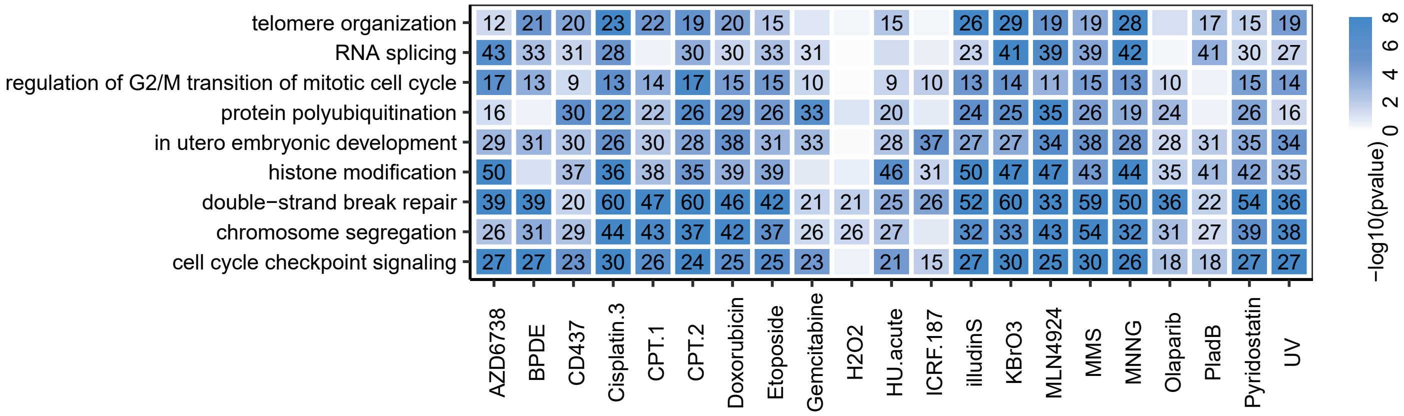

B

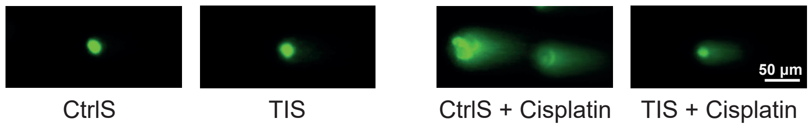

C

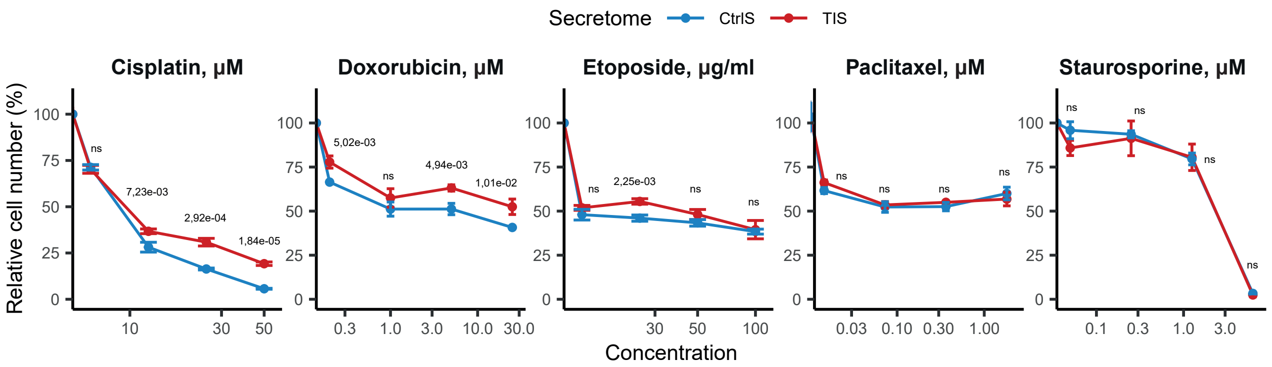

Supplementary Figure 6. Factors secreted by drug-stressed ovarian cancer cells protect recipient cancer cells from subsequent treatment with DNA-damaging drugs. ***(A)*** Heat map representation of the 21 CRISPR screens derived from data reported in [45]. The numbers in each screen indicate the number of genes which knockdown sensitize cells to different genotoxic agents. The gradient blue color scale represents -log10(p-values). BPDE - benzo(a)pyrene diol epoxide (helix distorting lesion); CD437 - anti-tumor toxin (DNA replication stress); Cisplatin.3 (inter- intrastrand crosslink/Helix distorting lesion); CPT.1 and CPT.2 - camptothecin (DNA strand breaks); Doxorubicin (DNA strand breaks); Etoposide (DNA strand breaks); Gemcitabine (DNA replication stress); H2O2 - Hydrogen Peroxide (Oxidative DNA damage); HU.acute - Hydroxyurea (DNA replication stress); ICRF.187 - TOP2 inhibitor; illudinS (transcription-interefering); KBrO3 (oxidative DNA damage); MLN4924 - Pevonedistat (NAE inhibitor); MMS - Methyl Metanesulfonate (base alkylation); MNNG (Base alkylation); Olaparib (PARP inhibitor) PladB - Pladienolide B (splicing-interfering); Pyridostatin (C-quadruplex stabilizer); UV - Ultraviolet Light (helix distorting lesion). ***(B)*** Representative comet assay images of SKOV3 cells stained with SYBR green after different types of treatment and observed under fluorescent microscope. SKOV3 cells were pre-incubated with therapy-induced (TIS) or control (CrlS) secretomes for 3 days (secretomes were collected as indicated in Fig. 3B) and then treated or untreated with cisplatin (10 μM) for 48 h. ***(C)*** Dose-response curves obtained by MTT assay of recipient SKOV3 cells incubated with therapy-induced (TIS) or control (CtrlS) secretomes for 3 days (secretomes were collected as indicated in Fig. 3B), and then treated with different concentrations of cisplatin, doxorubicin, etoposide, paclitaxel, or staurosporine for 2 days. The data represent the mean values ± SD (n=3 biologically independent experiments). The p-value was obtained by two-tailed unpaired Student’s t-test. Source data are provided as a Source Data file.

Extended Figure 7

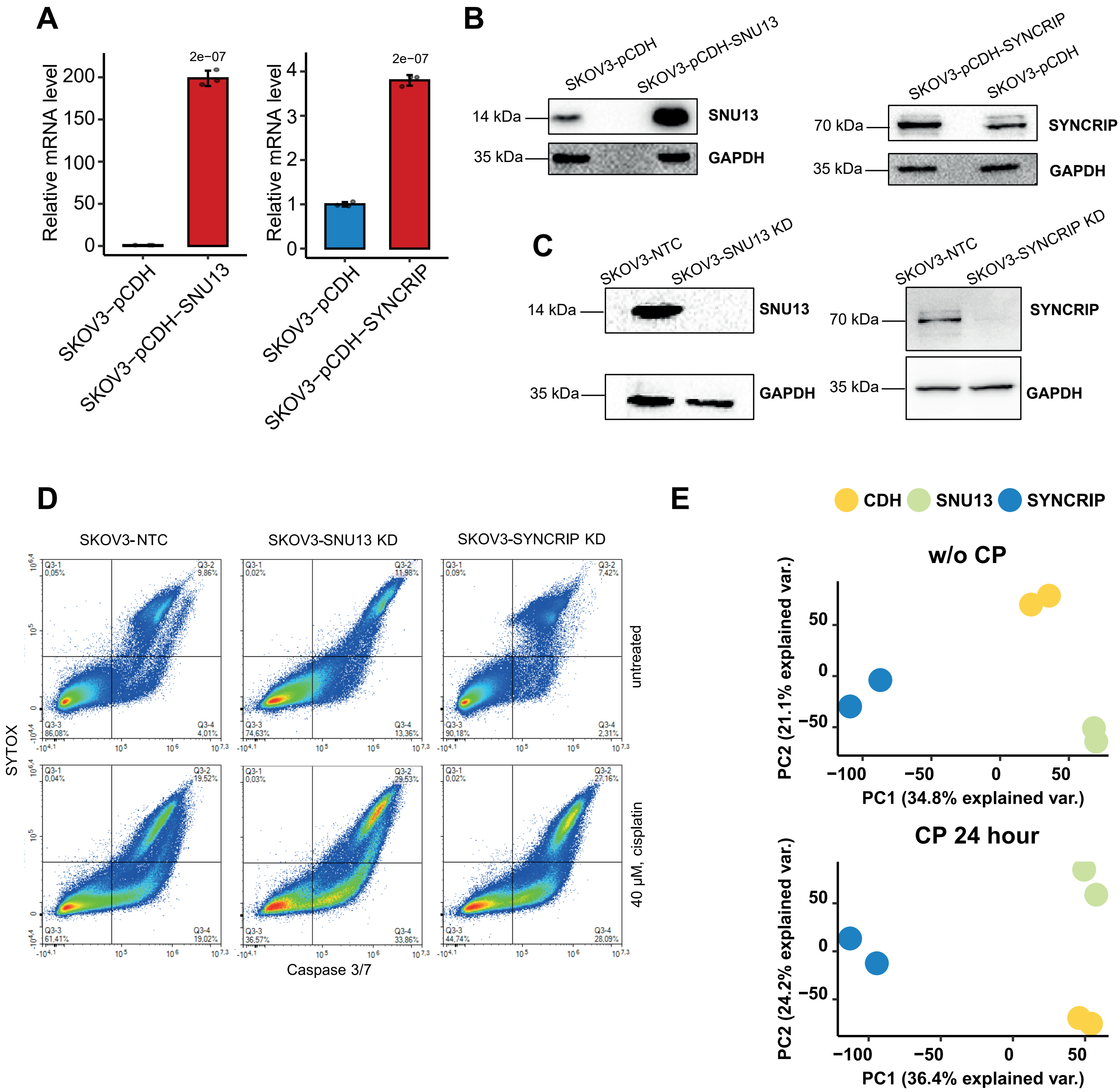

Supplementary Figure 7. Functional characterization of SKOV3 cells stably overexpressing SYNCRIP or SNU13 in the response to cisplatin. ***(A and B)*** RT-qPCR and western blotting analyses of SKOV3 cells stably expressing either SNU13, SYNCRIP or control empty vector pCDH. The RT-qPCR data represent the mean values ± SD (n=3 biologically independent samples). ***(C)*** Western blotting analysis of SKOV3 cells stably expressing shRNAs against *SNU13* (SKOV3-SNU13 KD), *SYNCRIP* (SKOV3-SYNCRIP KD) or control non-target shRNA (SKOV3-NTC). ***(D)*** Flow cytometry analysis of caspase 3/7 activity and SYTOX staining of SKOV3 cells stably expressing shRNAs against *SYNCRIP*, *SNU13* or control non-target shRNA (SKOV3-NTC) and treated with 40 μM of cisplatin for 24 hours. ***(E)*** The principal component analysis of RNAseq data for SKOV3 cells overexpressing SYNCRIP or SNU13 and control SKOV3-pCDH cells before (top panel) and 24 h after (bottom panel) cisplatin (40 μM) treatment. The p-value was obtained by two-tailed unpaired Student's t-test (*A*). Source data are provided as a Source Data file.

Extended Figure 8

A

Analogue of RNA	Sequence 5 '→ 3' (length, bases)
**U6atac**	GUGUUGUAUGAAAGGAGAGAAGGUUAGCACUCCCC UUGACAAGGAUGGAAGAGGCCCUCGGCCUGACA ACACGCAUACGGUUAAGGCAUUGCCACCUACUUCG UGGCAUCUAACCAUCGUUUUU **(125 bases)**
**U12**	GAUGCCUUAAACUUAUGAGUAAGGAAAAUAACGAU UCGGGGUGACGCCCGAAUCCUCACUGCUAAUGUG AGACGAAUUUUUGAGCGGGUAAAGGUCGCCCUCAA GGUGACCCGCCUACUUUGCGGGAUGCCUGGGAGU UGCGAUCUGCCCG **(150 bases)**
**GFP-89**	ACGUGCACGGCCACAAGUUCAGCGUGCGCGGCG AGGGCGAGGGCGACGCCGACUACGGCAAGCUGG AGAUCAAGUUCAUCUGCACCACC **(89 bases)**

B

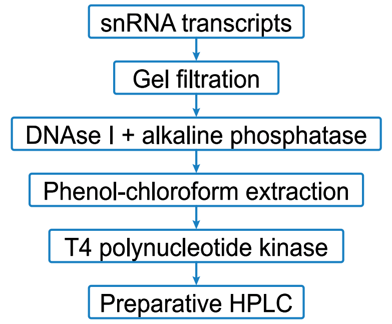

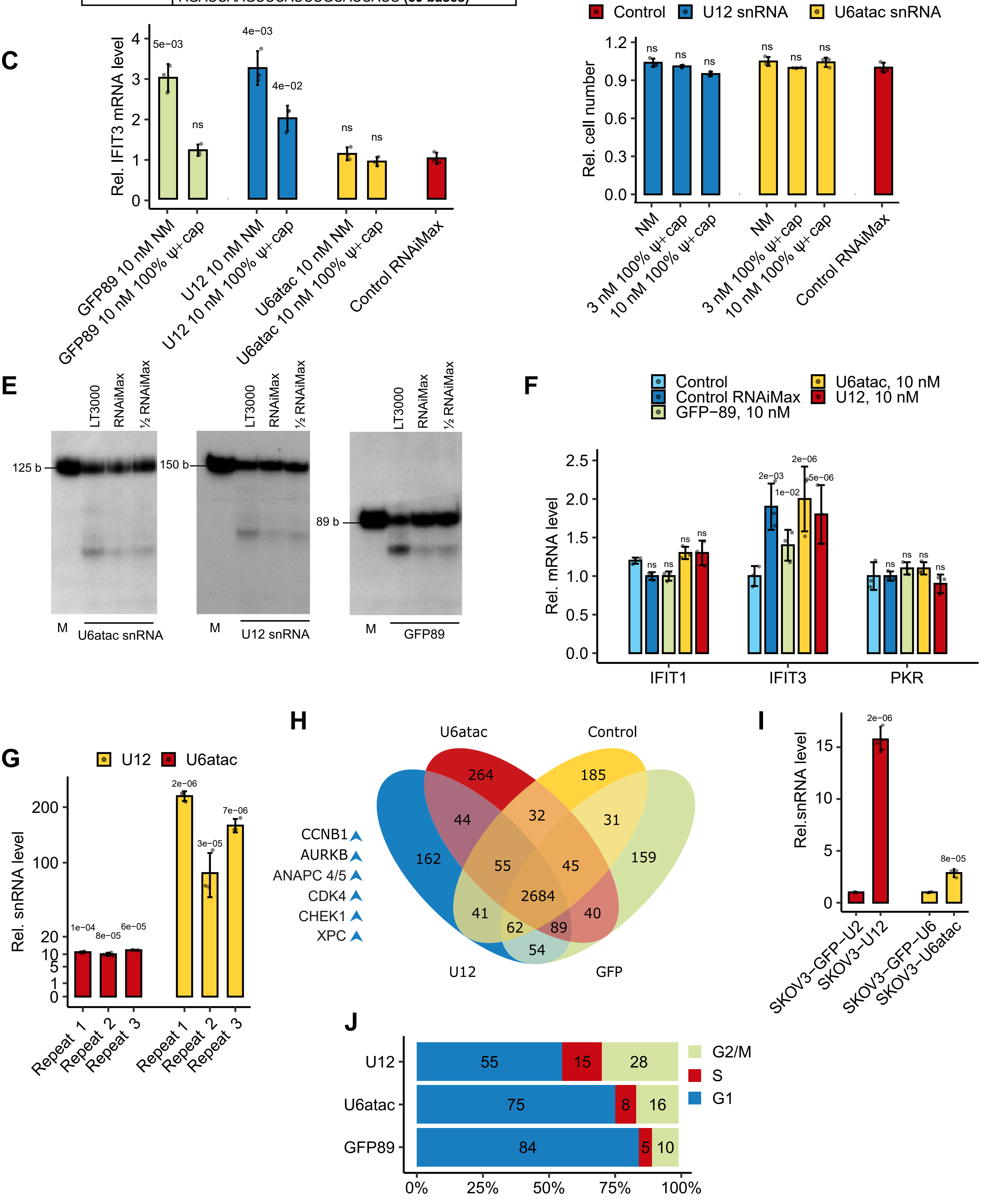

Supplementary Figure 8. Validation of snRNA transfection of SKOV3 cells. ***(A)*** The nucleotide sequences of U6atac snRNA, U12 snRNA, and a control mRNA fragment of the GFP (GFP89) used for *in vitro* synthesis. ***(B)*** Scheme of the *in vitro* synthesis of U6atac snRNA, U12 snRNA, and GFP89. ***(C)*** RT-qPCR data showing relative expression levels of interferon-regulated *IFIT3* in SKOV3 cells 48 h after transfection with non-modified (NM) or modified (100% pseudouridine (Ψ) and cap) analogs of U6atac snRNA, U12 snRNA, and GFP89. Control cells were incubated with empty lipofectamine RNAiMax. The data represent the mean values ± SD (n=3 biologically independent samples). ***(D)*** The viability of SKOV3 cells 48 h after transfection with 10 nM of non-modified (NM) and modified (100% pseudouridine (Ψ) and cap) U6atac snRNA, U12 snRNA or GFP89 with lipofectamine RNAiMax. Control cells were incubated with empty lipofectamine RNAiMax. The data represent the mean values ± SD (n=3 biologically independent experiments). ***(E)*** Autoradiography data reflecting the stability of U6atac snRNA, U12 snRNA, and GFP89 with radioactive labels in SKOV3 cells with different transfecting agents: Lipofectamine 3000 (LT 3000) or RNAiMax. SKOV3 cells were taken 48 h after transfection. M - molecular weight marker. ***(F)*** RT-qPCR data showing relative expression levels of interferon-regulated *IFIT1*, *IFIT3*, and *PKR* in SKOV3 cells 48 h after transfection with U6atac snRNA, U12 snRNA, or GFP89. Control cells were incubated with empty lipofectamine RNAiMax. All data represent the mean values ± SD (n=3 biologically independent samples). ***(G)*** RT-qPCR data showing the levels of U6atac and U12 snRNAs in SKOV3 cells 48 h after transfection with the corresponding snRNAs. Control cells were incubated with empty lipofectamine RNAiMax. All data represent the mean values ± SD (n=3 biologically independent samples). ***(H)*** Venn diagram representing the proteins identified in SKOV3 cells 48 h after transfection with 10 nM U6atac snRNA (red circle), U12 snRNA (blue circle), or GFP89 (green circle). Control cells (yellow circle) were incubated with empty lipofectamine RNAiMax. ***(I)*** RT-qPCR of SKOV3 cells stably expressing U6atac snRNA, U12 snRNA or control mRNA fragments of the GFP with different promoters: GFP-U2 (as a control for U12 snRNA) or GFP-U6 (as a control for U6atac snRNA). The RT-qPCR data represent the mean values ± the confidence interval (n=3 biologically independent samples). ***(J)*** Cell cycle analysis of SKOV3 cells 48 h after transfection with 10 nM U6atac snRNA, U12 snRNA, or GFP89.

The p-value was obtained by two-tailed unpaired Student's t-test (*C, D, F, G, I*). Source data are provided as a Source Data file.
