# Supplementary material for: Therapy-induced secretion of spliceosomal components mediates pro-survival crosstalk between ovarian cancer cells
Source: Nat Commun. 2024 Jun 19;15:5237. doi: 10.1038/s41467-024-49512-6 (PMC11187153; doi:10.1038/s41467-024-49512-6)
Supplement: Supplementary file 3 — Description of Additional Supplementary Files [file 41467_2024_49512_MOESM3_ESM.pdf]

Description of Additional Supplementary Files

Supplementary Data 1 - Information about patient samples and oligonucleotides
Sheet 1 – Detailed information about ovarian cancer patients
Sheet 2 – List of experiments with ovarian cancer ascitic fluids
Sheet 3 – List of oligonucleotides and their sequences used in this study
Sheet 4 – Determination of cisplatin in secretomes by LC-MS/MS

Supplementary Data 2 - Transcriptomic profiles of ovarian cancer cells isolated from paired cancer ascites before and after chemotherapy
Sheet 1 – Analysis of differentially expressed genes in ovarian cancer cells after chemotherapy
Sheet 2 – Results of the GSEA enrichment analysis of genes that were differentially expressed in ovarian cancer cells after chemotherapy

Supplementary Data 3 - Transcriptomic and proteomic profiles of ovarian cancer cells incubated with autologous ascitic fluids from patients before and after chemotherapy
Sheet 1 – Analysis of differentially expressed genes in epithelial ovarian cancer cells incubated with ascitic fluids after chemotherapy
Sheet 2 – Analysis of differentially expressed genes in neuroendocrine ovarian cancer cells incubated with ascitic fluids after chemotherapy
Sheet 3 – Proteins identified by LC-MS/MS analysis of ovarian cancer cells incubated with autologous ascitic fluids from patients before and after chemotherapy

Supplementary Data 4 - Proteome profiles of ovarian cancer ascitic fluids and in vitro secretomes before and after chemotherapy
Sheet 1 – Proteins identified by LC-MS/MS analysis of paired ovarian cancer ascitic fluids before and after chemotherapy
Sheet 2 – Proteins identified by LC-MS/MS analysis of secretomes from cisplatin-treated or untreated SKOV3 cells and Fibroblasts
Sheet 3 – Proteins identified by LC-MS/MS analysis of secretomes from cisplatin-treated or untreated cells
Sheet 4 – KEGG enrichment analysis of proteins whose abundance increased in therapy-induced secretomes
Sheet 5 – Table of spliceosomal proteins identified in ascitic fluids and secretomes after therapy
Sheet 6 – Comparison of proteins identified in ascitic fluids and secretomes with proteins related to stress granules and senescence-associated secretory phenotype

Supplementary Data 5 - Proteome and transcriptome profiles of SKOV3 cells in response to cisplatin treatment and incubation with secretomes before and after therapy
Sheet 1 – Proteins identified by LC-MS/MS analysis of SKOV3 cell fractions before and after cisplatin treatment
Sheet 2 – SILAC data analysis of unlabeled recipient SKOV3 cells after incubation with therapy-induced or control secretomes containing heavy-labeled proteins
Sheet 3 – Analysis of differentially expressed genes in SKOV3 cells incubated with therapy-induced secretomes
Sheet 4 – Analysis of differentially abundant proteins in SKOV3 cells incubated with therapy-induced secretomes
Sheet 5 – Analysis of differentially expressed genes in SKOV3 cells incubated with therapy-induced secretomes

Supplementary Data 6 - Transcriptome profiles of SKOV3 cells with overexpression of SYNCRIP or SNU13 in response to cisplatin treatment
Sheet 1 – Analysis of differentially expressed genes in SKOV3 with overexpression of SYNCRIP or SNU13 in response to cisplatin treatment

Supplementary Data 7 - Effect of extracellular spliceosomal snRNAs on the transcriptome and proteome of ovarian cancer cells
Sheet 1 – Analysis of differentially expressed genes in SKOV3 cells transfected with snRNAs
Sheet 2 – Proteins identified by LC-MS/MS analysis of SKOV3 cells transfected with snRNAs
