# Supplementary material for: Therapy-induced secretion of spliceosomal components mediates pro-survival crosstalk between ovarian cancer cells
Source: Nat Commun. 2024 Jun 19;15:5237. doi: 10.1038/s41467-024-49512-6 (PMC11187153; doi:10.1038/s41467-024-49512-6)
Supplement: Supplementary file 11 — Reporting Summary [file 41467_2024_49512_MOESM11_ESM.pdf]

nature portfolio

Corresponding author(s): Victoria O. Shender

Last updated by author(s): Apr 26, 2024

Reporting Summary

Nature Portfolio wishes to improve the reproducibility of the work that we publish. This form provides structure for consistency and transparency in reporting. For further information on Nature Portfolio policies, see our Editorial Policies and the Editorial Policy Checklist.

Statistics

For all statistical analyses, confirm that the following items are present in the figure legend, table legend, main text, or Methods section.

n/a	Confirmed	
☐	☒	The exact sample size (*n*) for each experimental group/condition, given as a discrete number and unit of measurement
☐	☒	A statement on whether measurements were taken from distinct samples or whether the same sample was measured repeatedly
☐	☒	The statistical test(s) used AND whether they are one- or two-sided<br>*Only common tests should be described solely by name; describe more complex techniques in the Methods section.*
☐	☒	A description of all covariates tested
☐	☒	A description of any assumptions or corrections, such as tests of normality and adjustment for multiple comparisons
☐	☒	A full description of the statistical parameters including central tendency (e.g. means) or other basic estimates (e.g. regression coefficient) AND variation (e.g. standard deviation) or associated estimates of uncertainty (e.g. confidence intervals)
☐	☒	For null hypothesis testing, the test statistic (e.g. *F*, *t*, *r*) with confidence intervals, effect sizes, degrees of freedom and *P* value noted<br>*Give P values as exact values whenever suitable.*
☒	☐	For Bayesian analysis, information on the choice of priors and Markov chain Monte Carlo settings
☐	☒	For hierarchical and complex designs, identification of the appropriate level for tests and full reporting of outcomes
☒	☐	Estimates of effect sizes (e.g. Cohen's *d*, Pearson's *r*), indicating how they were calculated

Our web collection on statistics for biologists contains articles on many of the points above.

Software and code

Policy information about availability of computer code

Data collection	Vendor programs for manipulations with Mass-spectrometers, Sequenators; PCR Real Time Amplificators; Flow Cytometer; Microscopes. This paper does not report the original code.
Data analysis	NovoExpress or FlowJo 10 software; CometScore pro (2.0.0.38) software; ImageJ software (version 2.9.0/1.54f) with FindFoci plugins; Image Lab 6.1 software; Salmon (version 1.4); R package “tximport”; R package “DESeq2”; R package of “FactoMineR”, “ggplot2”; bcl2fastq software (Illumina); “Trim Galore" (v.0.5.0); “Trimmomatic” (version 0.35); STAR (version 2.5.2b, version 2.7.6; https://code.google.com/archive/p/rna-star/); HTSeq-count (version 0.6.0; https://htseq.readthedocs.io/en/master/); R package “edgeR” (https://bioconductor.org/packages/release/bioc/html/edgeR.html); R package “sva”; MSConvert (ProteoWizard Software Foundation); ProteinPilot (version 4.5; SCIEX); MASCOT (version 2.5.1; http://www.matrixscience.com/); X! Tandem (ALANINE, 2017.02.01; http://www.thegpm.org/tandem/); Scaffold 4 (version 4.0.7; Proteome Software); MaxQuant (version 1.6.10.43; https://www.maxquant.org/); R package of “signatureSearch”; R/Bioconductor package “clusterprofiler”; R/Bioconductor package “ReactomePA”; Molecular Signatures Database (MSigDB); qbase+ (version 3.1; Biogazelle); Adobe Illustrator CC 2018. This paper does not report the original code.

For manuscripts utilizing custom algorithms or software that are central to the research but not yet described in published literature, software must be made available to editors and reviewers. We strongly encourage code deposition in a community repository (e.g. GitHub). See the Nature Portfolio guidelines for submitting code & software for further information.

Data

Policy information about availability of data

All manuscripts must include a data availability statement. This statement should provide the following information, where applicable:

- Accession codes, unique identifiers, or web links for publicly available datasets
- A description of any restrictions on data availability
- For clinical datasets or third party data, please ensure that the statement adheres to our policy

Publicly available datasets used in your study were obtained from the Gene Expression Omnibus (https://www.ncbi.nlm.nih.gov/geo/) database: GSE148003, GSE98559, GSE98230, GSE173201.
All RNAseq data generated in this study have been deposited in the Gene Expression Omnibus database (GEO) under the accession numbers:
[GSE241908] (Transcriptomic profiles of ovarian cancer cells isolated from paired cancer ascites before and after chemotherapy);
[GSE241909] (Transcriptomic profiles of ovarian cancer cells incubated with autologous ascitic fluids from patients before and after chemotherapy);
[GSE241910] (Effect of therapy-induced secretomes on the transcriptome of ovarian cancer cells);
[GSE241912] (Effect of extracellular spliceosomal snRNAs on the transcriptome of ovarian cancer cells);
[GSE241913] (Transcriptome of ovarian cancer cells overexpressing SYNCRIP or SNU13);
[GSE241914] (This SuperSeries is composed of the SubSeries listed upper) — and are publicly available as of the date of publication [https://www.ncbi.nlm.nih.gov/geo/query/acc.cgi?acc=GSE241914].
All proteomic datasets have been deposited to the ProteomeXchange Consortium via the PRIDE partner repository with the dataset identifiers:
1) [PXD019327] Proteome profiles of paired ovarian cancer ascites before and after chemotherapy [https://www.ebi.ac.uk/pride/archive/projects/PXD019327];
2)[PXD019096] Cisplatin-Induced Changes in Proteomic Profiles of Ovarian Cancer Cell and Fibroblast Secretomes [https://www.ebi.ac.uk/pride/archive/projects/PXD019096];
3) [PXD019642] Subcellular Relocalization of Proteins in Response to Cisplatin-Induced DNA Damage [https://www.ebi.ac.uk/pride/archive/projects/PXD019642];
4) [PXD027948] SILAC Strategy for Analysis of Proteins Released To the Extracellular Medium and Penetrated Into Recipient Cells [https://www.ebi.ac.uk/pride/archive/projects/PXD027948];
5) [PXD027950]) The Role of Extracellular Spliceosomal snRNAs in Communication of Ovarian Cancer Cells [https://www.ebi.ac.uk/pride/archive/projects/PXD027950];
6) [PXD027794] Effect of therapy-induced secretomes on the proteome of ovarian cancer cells [https://www.ebi.ac.uk/pride/archive/projects/PXD027794];
7) [PXD045647] Effect of malignant ascites before and after therapy on the proteome of ovarian cancer cells [https://www.ebi.ac.uk/pride/archive/projects/PXD045647];
8) [PXD045663] Cisplatin-Induced Changes in Proteomic Profiles of Ovarian Cancer Cell and HaCaT Secretomes [https://www.ebi.ac.uk/pride/archive/projects/PXD045663].
All processed data are available in the article, Supplementary files, and Source Data. Source Data are provided with this paper.

Research involving human participants, their data, or biological material

Policy information about studies with human participants or human data. See also policy information about sex, gender (identity/presentation), and sexual orientation and race, ethnicity and racism.

Reporting on sex and gender

All patients whose ascitic fluids were taken for this study were female because the object of our study is ovarian cancer, which represents gynecological disease.

Reporting on race, ethnicity, or other socially relevant groupings

All patients had Caucasian race.

Population characteristics

Pateint 1 - 45 y.o. small-cell carcinoma of the ovary (neuroendocrine tumor of the ovary), hypercalcemic type, stage III
Pateint 2 - 58 y.o. serous ovarian adenocarcinoma, stage IV
Pateint 3 - 68 y.o. serous ovarian adenocarcinoma, stage III
Pateint 4 - 50 y.o. serous ovarian adenocarcinoma, stage III
Pateint 5 - 57 y.o. serous ovarian adenocarcinoma, stage III
Pateint 6 - 49 y.o. serous ovarian adenocarcinoma, stage IV
Pateint 7 - 64 y.o. serous ovarian adenocarcinoma, stage IV
Pateint 8 - 55 y.o. serous ovarian adenocarcinoma, stage IIIC
Pateint 9 - 62 y.o. serous ovarian adenocarcinoma, stage III
Pateint 10 - 58 y.o. serous ovarian adenocarcinoma, stage III
Pateint 11 - 44 y.o. serous ovarian adenocarcinoma, stage III
Pateint 12 - 76 y.o. serous ovarian adenocarcinoma, stage IV
Pateint 13 - 59 y.o. serous ovarian adenocarcinoma, stage IV
Pateint 14 - 42 y.o. serous ovarian adenocarcinoma, stage III
Pateint 15 - 59 y.o. serous ovarian adenocarcinoma, stage III
Pateint 16 - 39 y.o. serous ovarian adenocarcinoma, stage III
Pateint 17 - 50 y.o. serous ovarian adenocarcinoma, stage III
Pateint 18 - 67 y.o. serous ovarian adenocarcinoma, stage IV
Pateint 19 - 48 y.o. serous ovarian adenocarcinoma, stage IIIC
Pateint 20 - 77 y.o. serous ovarian adenocarcinoma, stage III
Pateint 21 - 57 y.o. serous ovarian adenocarcinoma, stage IV
Pateint 22 - 50 y.o. serous ovarian adenocarcinoma, stage III
Pateint 23 - 65 y.o. serous ovarian adenocarcinoma, stage IIIC
Pateint 24 - 77 y.o. serous ovarian adenocarcinoma, stage III

	Pateint 25 - 68 y.o. mucinous ovarian adenocarcinoma, stage III<br>Pateint 26 - 57 y.o. serous ovarian adenocarcinoma, stage IIIC<br>Detailed information about each patient is provided in Supplementary Data 1.
Recruitment	Our patient selection criteria included a confirmed diagnosis of ovarian cancer and the presence of ascites.
Ethics oversight	All patients provided written informed consent for participation. Ethical approval was obtained from the Ethics Committees of the Russian Scientific Center of Roentgenoradiology (agreement and protocol no. 30-2018/E from 13 November 2018) and National Medical Research Center for Obstetrics, Gynecology, and Perinatology named after Academician V.I. Kulakov of the Ministry of Healthcare of the Russian Federation (protocol no. 10 of 5 December 2019).

Note that full information on the approval of the study protocol must also be provided in the manuscript.

Field-specific reporting

Please select the one below that is the best fit for your research. If you are not sure, read the appropriate sections before making your selection.

☒ Life sciences ☐ Behavioural & social sciences ☐ Ecological, evolutionary & environmental sciences

For a reference copy of the document with all sections, see nature.com/documents/nr-reporting-summary-flat.pdf

Life sciences study design

All studies must disclose on these points even when the disclosure is negative.

Sample size	The sample size was determined on the basis of our previous studies and the experience of the authors [Cancer Cell. 2018, 34(1): 119-135; Genome Med. 2018 Jun 27;10(1):49; Nat Commun. 2022 Oct 21;13(1):6246; Nat Cell Biol. 2022 Oct;24(10):1541-1557; Biomed Res Int. 2017;2017:3620510]. We expect changes in the gene/protein expression and function measurements to be detected with n=3 samples per group unless otherwise noted.
Data exclusions	No data were excluded from the analyses. All cells/animals that met proper experimental conditions were included in the analysis.
Replication	All experimental findings were repeated using multiple biological and technical replicates in each experiment. Precise numbers of biological replicates are given in the figure legend and in the "Methods" section. All experimental findings were reliably reproduced.
Randomization	For experiments, cells, biological samples, and animals used in this study were randomly assigned to their respective groups before the experiments were performed.
Blinding	No blinding was involved. The experimenters designed, performed and analyzed all experiments, so blinding is not applicable to these kinds of experiments. Majority of the results involved equipment-based quantitative measurements rather that subjective rating of the data that could be affected by no blinding.

Reporting for specific materials, systems and methods

We require information from authors about some types of materials, experimental systems and methods used in many studies. Here, indicate whether each material, system or method listed is relevant to your study. If you are not sure if a list item applies to your research, read the appropriate section before selecting a response.

Materials & experimental systems

n/a	Involved in the study	
☐	☒	Antibodies
☐	☒	Eukaryotic cell lines
☒	☐	Palaeontology and archaeology
☐	☒	Animals and other organisms
☒	☐	Clinical data
☒	☐	Dual use research of concern
☒	☐	Plants

Methods

n/a	Involved in the study	
☒	☐	ChIP-seq
☐	☒	Flow cytometry
☒	☐	MRI-based neuroimaging

Antibodies

Antibodies used	The following antibodies were used in the study (Supplier; Catalog number; Clone name/Lot; Dilution):<br>1. Anti-cisplatin modified DNA (Abcam, ab103261, GR263432-51, 1:200)<br>2. Anti-CD63 (Abcam, ab134045, GR3421999-3, 1:1000 for WB)<br>3. Anti-DHX9 (Abcam, ab183731, GR3271560-1, 1:10000)<br>4. Anti-GAPDH (HyTest, 5G4/5G4cc, L19/06, 1:1000)<br>5. Anti-HNRNPD (Cell Signaling Technology, #12382, 1:1000)<br>6. Anti-HNRNPM (Novus Biologicals, NB200-314, 16KL-2, 1:500)

7. Anti-HNRNPQ/R (Cell Signaling Technology, #8588, 1:500)
8. Anti-human SRSF4 (Novus Biologicals, NBP2-04144, A1, 1:2000)
9. Anti-Lamin B (Cloud-Clone Corp., PAF548Hu01, A20210708906, 1:1000)
10. Anti-mouse (Invitrogen, G-21040, 2122350, 1:40000)
11. Anti-mouse Alexa Fluor 488 (Thermo Fisher Scientific, A-11001, 2140660, 1:500)
12. Anti-rabbit Alexa Fluor 488 (Thermo Fisher Scientific, A-11008, 2284595, 1:500)
13. Anti-p53 (Cell Signaling Technology, #2524, 1:1000)
14. Anti-PRPF8 (Abcam, ab79237, 5 µg/ml for IP)
15. Anti-rabbit (Invitrogen, G-21234, 2156243, 1:40000)
16. Anti-mouse Alexa Fluor 555 (Thermo Fisher Scientific, A-28180, 1:500)
17. Anti-rabbit Alexa Fluor 555 (Thermo Fisher Scientific, A-21428, 1:500)
18. Anti-RPA32/RPA2 (phospho S33) (Abcam, ab211877, GR3365603-6, 1:500)
19. Anti-SF3B1 (Abcam, ab170854, GR125113-9)
20. Anti-SNU13 (Abcam, ab181982, GR149422-2, 1:1000)
21. Anti-SRSF1 (Invitrogen, #32-4500, TJ275391, 1:250)
22. Anti-SRSF2 (Abcam, ab204916, 1:1000)
23. Anti-SRSF3 (Abcam, ab 198291, GR3200532-3, 1:1000)
24. Anti-TIA1 (Invitrogen, MA5-26474, VC2967611, 1:2000)
25. Anti-U2AF1 (Abcam, ab172614, GR138325-8, 1:1000 for WB, 1:10 for IP)
26. Anti-U2AF65 (GeneTex, GTX55828, 822000219, 1:500)
27. Anti-γH2AX (Sigma-Aldrich, 05-636, JBW301, 1:200)
28. Anti-CD81-APC (BD Biosciences, #551112, 10 µl per probe)
29. Anti-Tubulin Beta (Cloud-Clone Corp., PAB870Hu01, A20210708905, 1:500)
30. Anti-Rad51 (Abcam, ab213, 1:200)
31. Anti-LIG1 (Abcam, ab177946, 1:1000)
32. Anti-TDP1 (Cell Signaling, #59710, 1:1000)
33. Anti-FOXM1 (Cell Signaling Technology, #5436, 1:1000)
34. Anti-Phalloidin Alexa 555 (Thermo Fisher Scientific, A-34055, 1:400)
35. Anti-CD63 (Santa Cruz Biotechnology; Cat. #sc-5275, 1:100 for IP)
36. Anti-CA125 (Novus Bio, NBP2-59023, MAB-02920, 1:50)
37. Anti-EpCam (Abcam, ab223582, GR3367015-9, 1:100)
38. Anti-CD44 (Sony, 2294010, 1:100)

Validation

All commercial antibodies were validated by the manufacturers as indicated on their web sites. Antibodies used for western blot were further validated by molecular weights of the target proteins. The validation statements of all antibodies are accessible to the public on the manufacturer's website:
1. https://www.nature.com/articles/s41419-020-03348-2
2. https://www.abcam.com/products/primary-antibodies/cd63-antibody-epr5702-late-endosome-marker-ab134045.html
3. https://www.abcam.com/products/primary-antibodies/rna-helicase-a-antibody-epr13521-ab183731.html
4. https://shop.hytest.fi/product/glyceraldehyde-3-phosphate-dehydrogenase-gapdh-antibody
5. https://www.cellsignal.com/products/primary-antibodies/auf1-hnrnp-d-d6o4f-rabbit-mab/12382
6. https://www.novusbio.com/products/hnrnp-m1-m4-antibody-1d8_nb200-314
7. https://www.cellsignal.com/products/primary-antibodies/hnrnp-q-r-d18b2-rabbit-mab/8588
8. https://www.novusbio.com/products/sfrs4-antibody_nbp2-04144
9. https://www.cloud-clone.com/products/PAF548Hu01.html
10. https://www.thermofisher.com/antibody/product/Goat-anti-Mouse-IgG-H-L-Cross-Adsorbed-Secondary-Antibody-Polyclonal/G-21040
11. https://www.thermofisher.com/antibody/product/Goat-anti-Mouse-IgG-H-L-Cross-Adsorbed-Secondary-Antibody-Polyclonal/A-11001
12. https://www.sigmaaldrich.com/NL/en/product/mm/16237?utm_source=google&utm_medium=cpc&utm_campaign=20859306919&utm_content=155908438319&gclid=Cj0KCQjwlN6wBhCcARIsAKZvD5iIlSg9MN3Gk61L1ie9L0wX3ALUkEa8oEpjKosyuktO_LU6VOl5migaAoFJEALw_wcB
13. https://www.cellsignal.com/products/primary-antibodies/p53-1c12-mouse-mab/2524
14. https://www.abcam.com/products/primary-antibodies/prpf8prp8-antibody-ab79237.html
15. https://www.thermofisher.com/antibody/product/Goat-anti-Rabbit-IgG-H-L-Cross-Adsorbed-Secondary-Antibody-Polyclonal/G-21234
16. https://www.thermofisher.com/antibody/product/Goat-anti-Mouse-IgG-H-L-Secondary-Antibody-Recombinant-Polyclonal/A28180
17. https://www.thermofisher.com/antibody/product/Goat-anti-Rabbit-IgG-H-L-Cross-Adsorbed-Secondary-Antibody-Polyclonal/A-21428
18. https://www.ncbi.nlm.nih.gov/pmc/articles/PMC7140816/
19. https://www.abcam.com/products/primary-antibodies/sf3b1-antibody-epr11987b-ab170854.html
20. https://www.abcam.com/products/primary-antibodies/nhp2l1-antibody-epr11671-ab181982.html
21. https://www.thermofisher.com/antibody/product/SRSF1-Antibody-clone-96-Monoclonal/32-4500
22. https://www.abcam.com/products/primary-antibodies/sc35-antibody-epr12238-ab204916.html
23. https://www.abcam.com/products/primary-antibodies/srsf3-antibody-epr16976-ab198291.html
24. https://www.thermofisher.com/antibody/product/TIA-1-Antibody-clone-OTI1D7-Monoclonal/MA5-26474
25. https://www.abcam.com/products/primary-antibodies/u2af35u2af1-antibody-epr12649b-ab172614.html
26. https://www.genetex.com/Product/Detail/U2AF65-antibody/GTX55828
27. https://www.sigmaaldrich.com/NL/en/product/mm/05636i?utm_source=google&utm_medium=cpc&utm_campaign=20859306919&utm_content=155908438319&gclid=Cj0KCQjwlN6wBhCcARIsAKZvD5jVnQ6mVQqJD5-ZQslU6VAGX1keinLJBLR-nmS23r4Z7iTBlkRZVdYaAi5aEALw_wcB
28. https://www.fishersci.com/shop/products/cd81-mouse-anti-human-apc-clone-js-81-bd/BDB551112
29. https://www.cloud-clone.com/products/PAB870Hu01.html
30. https://www.abcam.com/products/primary-antibodies/rad51-antibody-14b4-ab213.html
31. https://www.abcam.com/products/primary-antibodies/lig1-antibody-epr12464-ab177946.html

32. https://www.cellsignal.com/products/primary-antibodies/tdp1-d8d1b-rabbit-mab/59710
33. https://www.cellsignal.com/products/primary-antibodies/foxm1-d12d5-xp-rabbit-mab/5436
34. https://www.thermofisher.com/order/catalog/product/A34055
35. https://www.ncbi.nlm.nih.gov/pmc/articles/PMC8422976/
36. https://www.novusbio.com/products/ca125-muc16-antibody-cl2782_nbp2-59023
37. https://www.abcam.com/products/primary-antibodies/epcam-antibody-epr20532-225-ab223582.html
38. https://www.sonybiotechnology.com/us/catalog/product/view/id/5005/category/4/

Eukaryotic cell lines

Policy information about cell lines and Sex and Gender in Research

Cell line source(s)

All primary cultures of ovarian cancer cells were derived from paired ovarian cancer ascites from the same patients before and after chemotherapy (detailed information about biospecimens are given in Supplementary Table 1). Paired ascites samples were obtained from the National Medical Research Center for Obstetrics, Gynecology and Perinatology named after Academician V.I. Kulakov of the Ministry of Healthcare of the Russian Federation (Moscow, Russia) and from the Federal State Budgetary Institution Russian Scientific Center of Roentgenoradiology (RSCRR) of the Ministry of Healthcare of the Russian Federation (Moscow, Russia). Primary culture of human dermal fibroblasts were obtained from the Research and Clinical Center of Physical-Chemical Medicine. SKOV3, OVCAR3, Phoenix-GP cells were purchased from ATCC.
Eukaryotic cell lines:
1) Human ovarian cancer cell line SKOV3 (ATCC, HTB-77)
2) Human ovarian cancer cell line MESOV (ATCC, CLR-3272)
3) Human keratinocyte cells HaCaT (CLS, 300493)
4) Human ovarian cancer cell line OVCAR3 (ATCC, HTB-161)
5) hTERT FT282 cells (ATCC, CRL-3449)
6) Phoenix-GP packaging cell line (ATCC, CRL-3215)
Sub-lines:
1) SKOV3-pCDH
2) SKOV3-pCDH-SYNCRIP
3) SKOV3-pCDH-SNU13
4) SKOV3-pLKO.1-NTC
5) SKOV3-pLKO.1-SYNCRIP
6) SKOV3-pLKO.1-SNU13
7) SKOV3-lenti-U6atac
8) SKOV3-lenti-U6-GFP
9) SKOV3-lenti-U12
10) SKOV3-lenti-U2-GFP

Authentication

All cell lines were verified for purity using STR analysis.

Mycoplasma contamination

All cell lines were regularly tested for mycoplasma contamination. All cells were free from mycoplasma at the time of all experiments.

Commonly misidentified lines
(See ICLAC register)

None of the cell lines used are listed in the ICLAC list.

Animals and other research organisms

Policy information about studies involving animals; ARRIVE guidelines recommended for reporting animal research, and Sex and Gender in Research

Laboratory animals

6-8 weeks old female SHO-PRKDC SCID HR/HR1EW 43375 mice were used in this study. The mice were housed in groups of four animals per cage and had access to autoclaved water and pelleted feed. The cage environment was enriched with a mouse house. The mice were kept at a standard temperature of 22 C±2 C and relative humidity of 55% (45-70%) in a 12:12-hour light:dark cycle (lights on, 6 am to 6 pm).

Wild animals

The study did not involve wild animals.

Reporting on sex

We used female SHO-PRKDC SCID HR/HR1EW 43375 mice in this study, because females mice are more stable and less aggressive in terms of behavior despite the fact that they have the estrous cycle. Moreover, our object of our study is ovarian cancer, which represents gynecological disease.

Field-collected samples

The study did not involve samples collected from the field.

Ethics oversight

All animal experiments were carried out in compliance with the protocols and recommendations for the proper use and care of laboratory animals (EEC Directive 86/609/EEC). The study protocol was approved by the Committee on the Ethics of Animal Experiments of the Administration of SB RAS (Permit #40 from April, 4 2018).

Note that full information on the approval of the study protocol must also be provided in the manuscript.

Plants

Seed stocks	Studies involving plants were not conducted.
Novel plant genotypes	Studies involving plants were not conducted.
Authentication	Studies involving plants were not conducted.

Flow Cytometry

Plots

Confirm that:

☒ The axis labels state the marker and fluorochrome used (e.g. CD4-FITC).

☒ The axis scales are clearly visible. Include numbers along axes only for bottom left plot of group (a 'group' is an analysis of identical markers).

☒ All plots are contour plots with outliers or pseudocolor plots.

☒ A numerical value for number of cells or percentage (with statistics) is provided.

Methodology

Sample preparation	For apoptosis assay cells were stained with CellEvent Caspase-3/7 Green Flow Cytometry Assay Kit (ThermoFisher) according to the manufacturer's protocol. Untreated cells were used as a negative control.<br>For cell cycle analysis, cells were fixed in 300 µl ice-cold 70% EtOH, while vortexing slightly, incubated at -20°C for at least 2 hours, washed with 3 ml of PBS, and incubated with 0.1% Triton X-100 in PBS supplemented with 4',6-diamidino-2-phenylindole (DAPI) (1 µg/ml) (Invitrogen, # 62248) for 30 min.<br>To analyze CD9-positive EVs, they were immune-selected with Exosome-Human CD9 Flow Detection Reagent (ThermoFisher Scientific; #10620D) according to the manufacturer protocol. CD9 Deanabeads (15 µl per probe) were incubated with collected EVs overnight at 4°C. After incubation, EVs-coated magnetic beads were washed with 0.05% Tween 20 / PBS buffer. Some of the samples were stained against a specific protein marker of exosome antiCD81-APC (BD Biosciences, #551112, 10 µl per probe) for 2 hours on a rotator at RT followed by flow analysis with BD LSRFortessa™ Cell Analyzer (BD, USA). Population of immunoisolated EVs are CD81 positive (data not shown). Red (PE channel) and green (FITC channel) fluorescence of the EVs-coated beads were registered for protein SRCF4-RFP and SYNCRIP-GFP or SNU13-GFP detection, correspondingly. The data has been processed with Kaluza software (Beckman Coulter, USA).
Instrument	NovoCyte Flow Cytometer (ACEA biosciences), BD LSRFortessa™ Cell Analyzer (BD).
Software	NovoExpress or FlowJo 10 software, Kaluza software.
Cell population abundance	All cells were analyzed without separation into different populations.
Gating strategy	The samples were gated by FSC-H and SSC-H to distinguish cells from debris.

☒ Tick this box to confirm that a figure exemplifying the gating strategy is provided in the Supplementary Information.
